# Immune response to SARS-CoV-2 mRNA vaccination in multiple sclerosis patients after rituximab treatment interruption

**DOI:** 10.3389/fimmu.2023.1219560

**Published:** 2023-07-27

**Authors:** Remigius Gröning, Andy Dernstedt, Clas Ahlm, Johan Normark, Peter Sundström, Mattias N. E. Forsell

**Affiliations:** ^1^ Department of Clinical Microbiology, Umeå University, Umeå, Sweden; ^2^ Molecular Infection Medicine Sweden (MIMS), Umeå University, Umeå, Sweden; ^3^ Wallenberg Centre for Molecular Medicine (WCMM), Umeå University, Umeå, Sweden; ^4^ Department of Clinical Sciences, Neurosciences, Umeå University, Umeå, Sweden

**Keywords:** multiple sclerosis, vaccination, COVID-19, rituximab, B cell immunology

## Abstract

Peripheral B cell depletion *via* anti-CD20 treatment is a highly effective disease-modifying treatment for reducing new relapses in multiple sclerosis (MS) patients. A drawback of rituximab (RTX) and other anti-CD20 antibodies is a poor immune response to vaccination. While this can be mitigated by treatment interruption of at least six months prior to vaccination, the timing to resume treatment while maintaining subsequent vaccine responses remains undetermined. Here, we characterized SARS-CoV-2 S-directed antibody and B cell responses throughout three BNT162b2 mRNA vaccine doses in RTX-treated MS patients, with the first two doses given during treatment interruption. We examined B-cell mediated immune responses in blood samples from patients with RTX-treated MS throughout three BNT162b2 vaccine doses, compared to an age- and sex-matched healthy control group. The first vaccine dose was given 1.3 years (median) after the last RTX infusion, the second dose one month after the first, and the third dose four weeks after treatment re-initiation. We analyzed SARS-CoV-2 S-directed antibody levels using enzyme-linked immunosorbent assay (ELISA), and the neutralization capacity of patient serum against SARS-CoV-2 S-pseudotyped lentivirus using luciferase reporter assay. In addition, we assessed switched memory (CD19^+^CD20^+^CD27^+^IgD^-^), unswitched memory (CD19^+^CD20^+^CD27^+^IgD^+^), naïve (CD19^+^CD20^+^CD27^-^IgD^+^), and double negative (DN, CD19^+^CD20^+^CD27^-^IgD^-^) B cell frequencies, as well as their SARS-CoV-2 S-specific (CoV^+^) and Decay Accelerating Factor-negative (DAF^-^) subpopulations, using flow cytometry. After two vaccine doses, S-binding antibody levels and neutralization capacity in SARS-CoV-2-naïve MS patients were comparable to vaccinated healthy controls, albeit with greater variation. Higher antibody response levels and CoV^+^-DN B cell frequencies after the second vaccine dose were predictive of a boost effect after the third dose, even after re-initiation of rituximab treatment. MS patients also exhibited lower frequencies of DAF^-^ memory B cells, a suggested proxy for germinal centre activity, than control individuals. S-binding antibody levels in RTX-treated MS patients after two vaccine doses could help determine which individuals would need to move up their next vaccine booster dose or postpone their next RTX infusion. Our findings also offer first indications on the potential importance of antigenic stimulation of DN B cells and long-term impairment of germinal centre activity in rituximab-treated MS patients.

## Introduction

Multiple sclerosis (MS) is an autoimmune disease that causes inflammation of the central nervous system (1). Although the underlying cause remains unknown, disease susceptibility is increased through a combination of heritable and environmental factors (2). Traditional disease modifying treatments (DMT), such as broad immunosuppressive and –modulatory drugs have a positive impact on the relapse rate of MS. However, their effect on disability advancement is limited during progressive MS (3). The suspected involvement of B cells in MS pathophysiology was proven in experimental trials using treatments specifically targeting B cells (1, 4). Rituximab (RTX) is a genetically engineered chimeric monoclonal antibody that depletes CD20^+^ cells, including most B cell subsets except CD20^-^ plasma cells. Accordingly, RTX has been shown to be highly effective to reduce inflammatory brain lesions and clinical relapses for a prolonged period (4, 5). Therefore, the treatment has been introduced as standard-of-care for MS patients at many hospitals in Sweden and elsewhere (6). The inhibition of the B cell-dependent adaptive immune system by RTX and other DMTs has also been shown to lead to a higher rate of hospitalization due to COVID-19 (7–10).

Due to its B cell-depleting effect, RTX treatment is detrimental for vaccine-induced responses. Treatment interruption is therefore required to mount efficient vaccine-induced responses, as has been shown (11, 12). In the case of MS patients, non-live COVID-19 vaccines, like the mRNA vaccine BNT162b2, were demonstrated safe and effective (13). At Umeå University Hospital, MS patients were under RTX treatment interruption prior to the first dose of BNT162b2, then resumed RTX approximately four weeks after the second vaccine dose. Here we studied B cell responses to repeated vaccination with BNT162b2 during RTX treatment interruption, with the goal to identify factors that could predict the efficacy of booster vaccinations after RTX treatment re-initiation.

## Patients and methods

### Patient cohort and material

**Figure 1:**
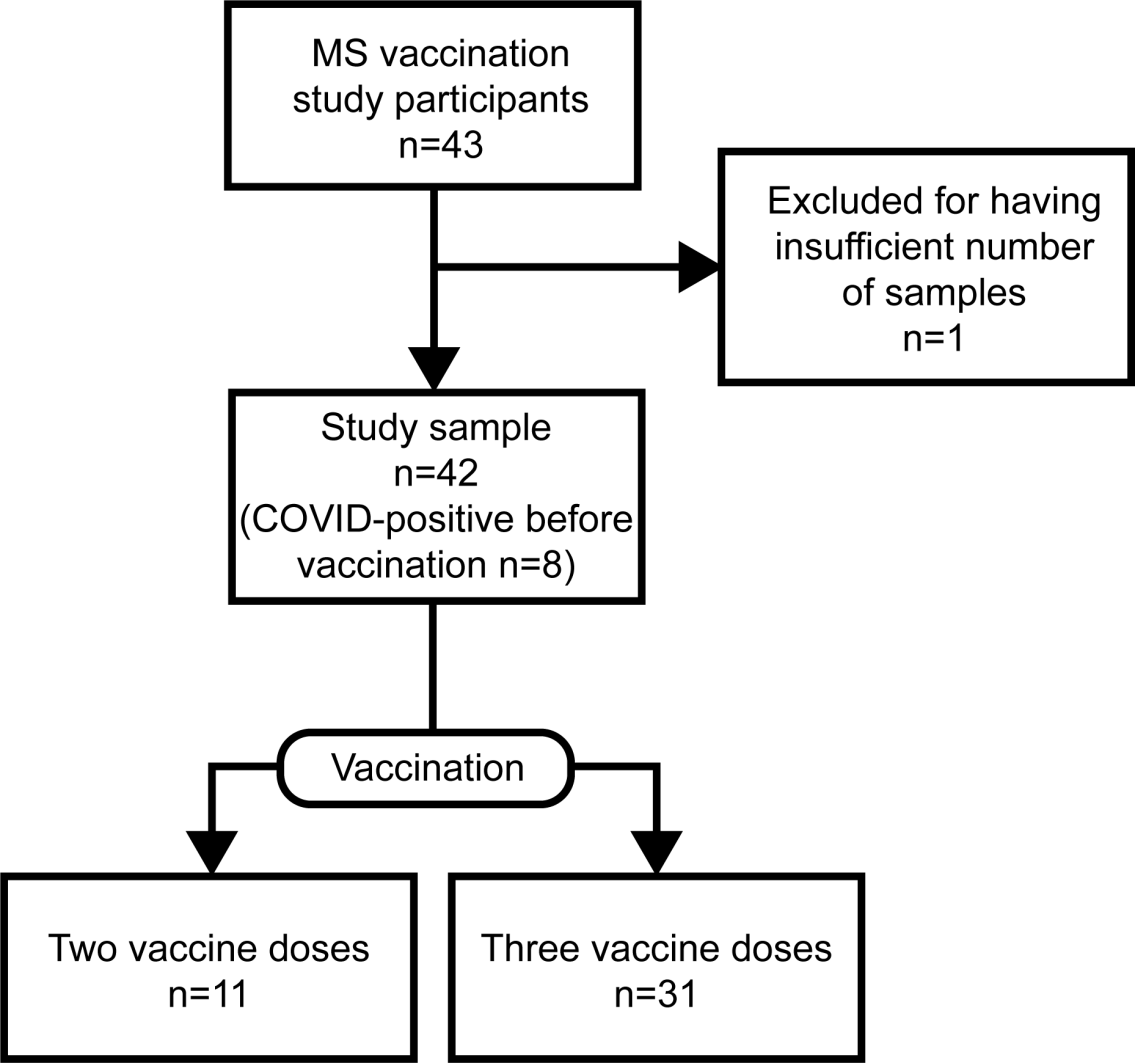
Inclusion flowchart of study cohort. Depicted are the number of vaccinated rituximab-treated (RTX) multiple sclerosis (MS) patients participating in the study and the final study sample after exclusion. The study sample is characterized further by the number of vaccine doses and COVID-experienced participants.

We enrolled 43 MS patients *via* the Neurological Department at Umeå University Hospital after informed consent, of which 42 had sufficient samples drawn for a longitudinal study (**Tables 1**, **2**; **Figure 1**). All had relapsing-remitting disease and were at present exclusively treated with RTX. Eight out of 43 individuals (19%) contracted COVID-19 before vaccination. All were vaccinated with Pfizer-BioNTech’s BNT162b2 mRNA vaccine half to two-and-a-half years after their last RTX infusion. The control cohort comprised 20 age- and sex-matched healthy controls, that were vaccinated with BNT162b2 (**Tables 1**, **2**). For the antibody avidity assay, we studied a different control cohort consisting of 10 individuals, which received three doses of BNT162b2.

**Table 1:** Clinical characteristics of 43 relapsing-remitting multiple sclerosis cases treated with rituximab and 20 non-MS control individuals.

Characteristics	All MS patients (N=43)	Non-MS control group (N=20)
Female n (%)	26 (60)	12 (60)
Age at disease onset	26 (15-47)	
Years of disease duration	9 (1-25)	
Age at enrollment	38 (25–64)	41.5 (20-59)
BMI	25 (19-43)	23.6 (18.7-41.6)
EDSS	1.0 (0-4.0)	
Covid 19 before vaccination n	8	0
Clinical parameters∗		
B cell count (cells/µl)	123 (1-817)	
Vitamin D level (nmol/l)	85 (54-149)	
Lymphocyte count (10^9^/l)	1.9 (1-3.4)	
CRP (mg/ml)	1.3 (0.6-20)	
Comorbidities	None (n=29), asthma (n=7), hypertension (n=2), hypothyroidism (n=2), diabetes type II (n=1), obesity (n=1), hyperlipidemia (n=1), prolactinoma (n=1), activated protein C resistance (n=1)	None (n=5), allergy (n=1), asthma (n=5), vaso-occlusive crisis (n=2), diabetes type I (n=1), gastritis (n=1), IgA nephritis (n=1), migraine (n=2)

All figures are median (range) unless stated otherwise. BMI, body mass index; CRP, c-reactive protein; DMD, disease modifying drug; EDSS, Extended Disability Status Scale. **∗**B cell (CD19+) count, vitamin D levels (25[OH]D), lymphocyte count, and CRP from the latest sample preceding rituximab treatment re-initiation.

**Table 2:** Rituximab treatment characteristics of 43 relapsing-remitting multiple sclerosis cases.

Rituximab treatment	All MS patients (N=43)
Number of rituximab infusions before vaccine dose 1	8 (1-11)
Rituximab total dosage (mg) before vaccine dose 1	5500 (1000-10500)
Rituximab last infusion dose (mg)	500 (300-1000)
Years since last rituximab infusion at vaccine dose 1	1.3 (0.52-2.6)
DMD before rituximab n (%)	
None	19 (44)
Interferon beta	19 (44)
Natalizumab	14 (33)
Dimethyl fumarate	1 (2.3)
Glatiramer acetate	2 (4.7)
Fingolimod	3 (7.0)

All figures are median (range) unless stated otherwise.

According to a consensus document by the Swedish MS-association regarding general vaccinations for MS patients on DMTs (14), RTX retreatment was postponed until one month after two BNT162b2 doses, with the first dose given when the B cell count exceeded 20 cells/µl and the second subsequently one month after. Thus, RTX treatment resumed before the third vaccine dose.

In case of the rituximab-treated (RTX) multiple sclerosis (MS) group, blood samples were collected as depicted in **Supplementary Figure 1**. Out of eight individuals, which had COVID-19 before vaccination, two got only one vaccine dose. Their sampling timepoints after the first dose were defined as after second dose, in order to compare them with the remaining individuals. Regarding the non-MS control cohort, blood samples were collected from 19 individuals before the first vaccine dose, 19 at one week after the second, 19 at one month after the second, 17 at two to three months after the second, and 15 at four to eight months after the second.

For the avidity assay, blood samples for the control group were collected from 10 individuals at one week after the second vaccine dose, two to three months after the second, four to eight months after the second, and one to two months after the third. Samples for the RTX-MS group were collected from 10 individuals as a subgroup of the 43 individuals examined throughout the rest of the study. For this group, 10 samples were collected at one week after the second vaccine dose, two to three months after the second, one to two months after the third, and 5 samples at four to eight months after the second.

8 mL of blood was collected in BD Vacutainter sodium-heparin CPT tubes (362780, BD, Franklin Lakes, NJ) and serum gel tubes (366566, BD) for the isolation of peripheral blood mononuclear cells (PBMCs) and serum, respectively, according to manufacturer’s recommendations. Briefly, CPT tubes were centrifuged at 2000xg for 20 min at RT, and cell interphase layer was collected and washed in PBS, followed by cryopreservation in FBS with 10% DMSO and stored in liquid N2 until flow cytometric analysis. Lymphocyte count, flow cytometry B-cell count (CD19^+^ cells), 25(OH)D, and c-reactive protein (CRP) routinely analyzed at the hospital laboratory were retrieved from the last blood sample before the first vaccine dose. The last estimate of MS disability was assessed using Extended Disability Status Scale (EDSS) (15).

### Standard protocol approvals, registrations, and patient consents

All patients provided written informed consent. The study was part of a clinical trial to study immunogenicity of COVID-19 vaccination (EudraCT 2021-000683-30, ClinicalTrials.gov ID NCT04920357). Ethical approvement was obtained by the Swedish Ethical Review Authority (Dnr 2021-00055 and subsequent approved amendments).

### Production of SARS-CoV-2-S protein and -pseudotyped lentivirus

The 2019-nCoV S protein was produced using the Gibco ExpiCHO Expression System (Thermo Fisher Scientific, Waltham, MA) and purified as previously described in Byström et al. (16).

A lentivirus containing the luciferase reporter gene and truncated 2019-nCoV-D614G-S protein was produced with the FuGENE 6 transfection reagent (Promega, Madison, WI) according to the manufacturer’s protocol. 19.5 million HEK 293T cells (CRL-3216, ATCC, Manassas, VA) were seeded per T175 flask in Dulbecco’s Modified Eagle Medium (DMEM) (Thermo Fisher Scientific) + 10% fetal bovine serum (FBS) + 1000 U/L Penicillin+Streptomycin (D10 medium) for SARS-CoV-2-pseudotyped lentivirus production and incubated at 37°C and 5% CO_2_. On the next day, 109 µL of Fugene 6 (Promega) and 1100 µL of Opti-MEM (Thermo Fisher Scientific) per T175 flask was mixed in a sterile polystyrene tube and incubated for 5 min at room temperature (RT). 21.1 µg of pHR’ CMV Luc (luciferase reporter gene), 21.1 µg of pCMV delta8.2 (lentivirus backbone), 0.37 µg of TMPRSS2 (all provided by Drs. Barney Graham and Kizzmekia Corbett at the Vaccine Research Center, Bethesda, MD), and 1.2 µg of pHDM SARS-CoV-2 Spike D614G 19-C-terminal deletion plasmid DNA (provided by Daniel Sheward, Karolinska Institute, Solna, Sweden) was added per T175 flask to the previous mixture and incubated for 15 min at RT. Finally, the reagent-DNA mixture was added to the medium in the T175 flask containing the 293T cells and the flask returned to the incubator.

On the subsequent day, the medium in the T175 flask was exchanged with 19 mL of fresh D10 medium per flask and the flask returned to the incubator. On the following day, the cell supernatant was harvested in 50 mL tubes and centrifuged at 210xg for 5 min and passed through a 0.45 µm PVDF sterile filter (Merck Millipore, Burlington, MA). Finally, the supernatant was aliquoted in 1 mL sample volume and stored at -80°C.

### Titration of pseudotyped lentivirus

HEK293T-hACE2.MF cells (provided by Drs. Barney Graham and Kizzmekia Corbett at the Vaccine Research Center) were seeded at a density of 5000 cells per well in a 96-well black cell culture plate (Greiner, Kremsmünster, Austria) in Dulbecco’s Modified Eagle Medium (DMEM) (Thermo Fisher Scientific) + 10% fetal bovine serum (FBS) + 1000 U/L Penicillin+Streptomycin (D10 medium) and incubated at 37°C, 5% CO_2_. On the next day, the pseudotyped lentivirus was thawed and diluted in a 96-well deep plate (Corning) in the following manner. 1:2 dilutions of pseudovirus with Minimum Essential Medium (MEM, Thermo Fisher Scientific) + 5% FBS (MEM5 medium) were prepared in the 11 wells of the first row. The last well contained only MEM5 medium. The dilution plate was incubated for 45 min at 37°C. The medium from the cells prepared on the previous day was removed and 50 µL of pseudovirus dilution was added to the cells in 8 replicates and incubated for 2 h at 37°C, 5% CO_2_. Afterwards, 150 µL of MEM5 medium was added to each well and incubated for 3 days at 37°C, 5% CO_2_.

After 3 days, cells were lysed, and luciferase reagent added according to the manufacturer’s instructions (Luciferase Assay System, Promega). The luminescence was measured on a Tecan Sunrise microplate reader within 2 minutes of adding the reagent with 1 second integration time. A pseudovirus dilution step for the neutralization assay was selected that yielded 1000-times the luminescence value of the medium-only wells.

### Neutralization assay

Neutralization of the authentic SARS-CoV-2 virus was conducted as previously described in Byström et al. (16). For the neutralization of the pseudotyped lentivirus, serum samples were inactivated for 20 min at 56°C. 5000 cells of 293T-hACE2.MF per well were seeded in a 96-well black cell culture plate (Greiner) in D10 medium and incubated at 37°C, 5% CO_2_. The next day, for each serum sample an 8-point 3-fold dilution series of the sample was prepared in a V-bottom 96-well plate in MEM5 medium. Then, pseudovirus diluted at half the chosen dilution was added to each serum dilution point at 1:1 ratio. Four wells per cell plate contained MEM5 medium, as well as four wells with the chosen pseudovirus dilution. The dilution plate was incubated for 45 min at 37°C. Afterwards, the medium was removed from the cells and 50 µL of the serum sample-pseudovirus mix was added in duplicates and incubated for 2 h at 37°C, 5% CO_2_. Afterwards, 150 µL of M5 medium was added to each well and incubated for 3 days at 37°C, 5% CO_2_. After 3 days the plates were prepared and measured as described in the previous paragraph.

### SARS-CoV-2 spike-specific enzyme-linked immunosorbent assay and avidity assay

The ELISA was conducted as previously described in Byström et al. (16), with patient serum samples diluted 1/50 in blocking buffer, and seven additional 5-fold serial dilutions.

For the avidity assay, the dilution series was prepared in duplicates. Before the addition of the conjugate antibody, the second replicate of the dilution series was incubated with 1.5 M NaSCN in PBS for 10 min at RT and the plates subsequently washed. The avidity index was determined by the AUC of the NaSCN-treated replicate divided by the AUC of the untreated replicate.

### Flow cytometric characterization of B cells

Isolated Purified SARS-CoV-2 spike protein was tetramerised with Strep-Tactin conjugated to APC and PE (IBA Lifesciences, Göttingen, Germany), respectively, for a minimum of 20 minutes at 4°C protected from light. Cryopreserved PBMCs were thawed in a 37°C water bath, washed in sterile PBS supplemented with 2% FBS (PBS+FBS), and transferred to a V-bottom 96-well plate for staining. Unless otherwise specified, all staining steps of the cells were performed at 4°C protected from light. 25nM each of APC and PE conjugated protein were incubated on the PBMCs for 60 mins. Further, cells were stained with 1:1000-diluted Zombie NIR (Biolegend, San Diego, CA) according to manufacturer’s instructions. Antibodies targeted towards surface antigens (**Table 3**) were diluted in PBS+FBS and incubated with cells for 25 min. Cells were fixed with eBioscience FoxP3/Transcription Factor Staining Buffer Set (Thermo Fisher Scientific), according to manufacturer’s instructions. Finally, cells were resuspended in sterile PBS+FBS and strained through 70µm pre-separation filters (Miltenyi Biotec, Bergisch Gladbach, Germany) before acquisition on a ZE5 flow cytometer (BioRad, Hercules, CA). The acquisition speed was 1 µl/second and sample temperature 4°C during acquisition. FCS files were analysed using FlowJo v10. Total B cells were defined as viable CD19^+^CD20^+^, and memory populations were distinguished by their expression of CD27 and IgD (17, 18).

**Table 3:** List of flow cytometric antibodies used.

Antigen	Fluorophore	Clone	Vendor
CD19	BUV496	SJ25C1	BD
CD20	AlexaFluor-700	2H7	Biolegend
CD27	BV421	MT271	BD
IgD	BV510	IA6-2	BD
DAF	PE-Cy7	JS11	Biolegend
CD3	BV510	SK7	Biolegend
CD4	BUV395	SK3	BD
CCR7	BV421	2-21-A	BD
CD45RA	BV786	HI100	BD
CD25	BV711	2A3	BD
DAF	APC	JS11	Biolegend

### Statistical analysis

For the neutralization assay, all values were subtracted by the average value of the medium-only wells and divided by the average of pseudovirus-only wells. The serum dilution required to reduce the pseudovirus infection by 50% (ID50) was determined with GraphPad Prism 9 (GraphPad Software, San Diego, CA). For the ELISA, all values were subtracted by the average value of the blocking buffer-only wells. Area under the curve (AUC) of the dilution curve was determined with GraphPad Prism 9 (GraphPad Software), with a baseline level of 0.2.

A mixed model with Geisser-Greenhouse correction and matched timepoints per individual was used together with Tukey’s multiple comparisons test for comparing S-binding IgG and neutralizing antibody levels between separate groups and time points. A Spearman correlation test was used for correlation analysis of multiple serology, clinical, and demographic data. All analysis was done in Graphpad Prism 9.0 (GraphPad Software).

Parameters chosen for Spearman correlation: AUC of SARS-CoV-2 spike-specific antibody levels one week after the second vaccine dose, ID50 SARS-CoV-2-pseudotyped lentivirus neutralization capability one week after the second vaccine dose, SARS-CoV-2 spike-specific antibody levels one week after the third vaccine dose, SARS-CoV-2-pseudotyped lentivirus neutralization capability one week after the third vaccine dose, and foldchange of SARS-CoV-2 spike-specific antibody levels after and before third vaccine dose, days since last RTX treatment before first vaccine dose, days since MS disease onset before first vaccine dose, days since last RTX treatment before third vaccine dose, total administered RTX amount since treatment start [mg], blood vitamin D concentration before first vaccine dose [nmol/L], blood total lymphocyte count before first vaccine dose [10^9 cells/L], blood C-reactive protein levels before first vaccine dose [mg/mL], blood total B cell count before first vaccine dose [cells/µL], age, Body Mass Index, and frequencies of B cell populations [%] one month after the second vaccine dose (CD27+IgD-, SARS-CoV-2+CD27+IgD-, CD27-IgD-, SARS-CoV-2+CD27-IgD-).

For the flow cytometry results, comparisons within groups were done by Wilcoxon matched-pairs signed rank test, and comparisons between groups by Mann-Whitney’s test, using Graphpad Prism 9.0 (GraphPad Software).

## Results

### Higher titers of neutralizing antibodies in COVID-experienced than COVID-naïve MS patients after vaccination

Of the 43 study participants in the MS group, we were able to acquire longitudinal samples from 42 individuals (**Figure 1**). Of these, all had been diagnosed with relapsing-remitting MS and been on RTX treatment interruption for a median of 1.3 years prior to BNT162b2 vaccination (**Tables 1**, **2**). This allowed for reconstitution of peripheral B cells above 20 cells/µL prior to receiving the 1^st^ vaccine dose. Additional demographic and clinical data for the MS patients included in this study are shown in **Tables 1**, **2**. RTX treatment was resumed for all patients at four weeks after the second dose of BNT162b2. Eight out of 43 individuals (19%) contracted COVID-19 before vaccination but after treatment interruption. Blood sampling of patients in the context to the three vaccine doses is described in detail in **Supplementary Figure 1**.

**Figure 2:**
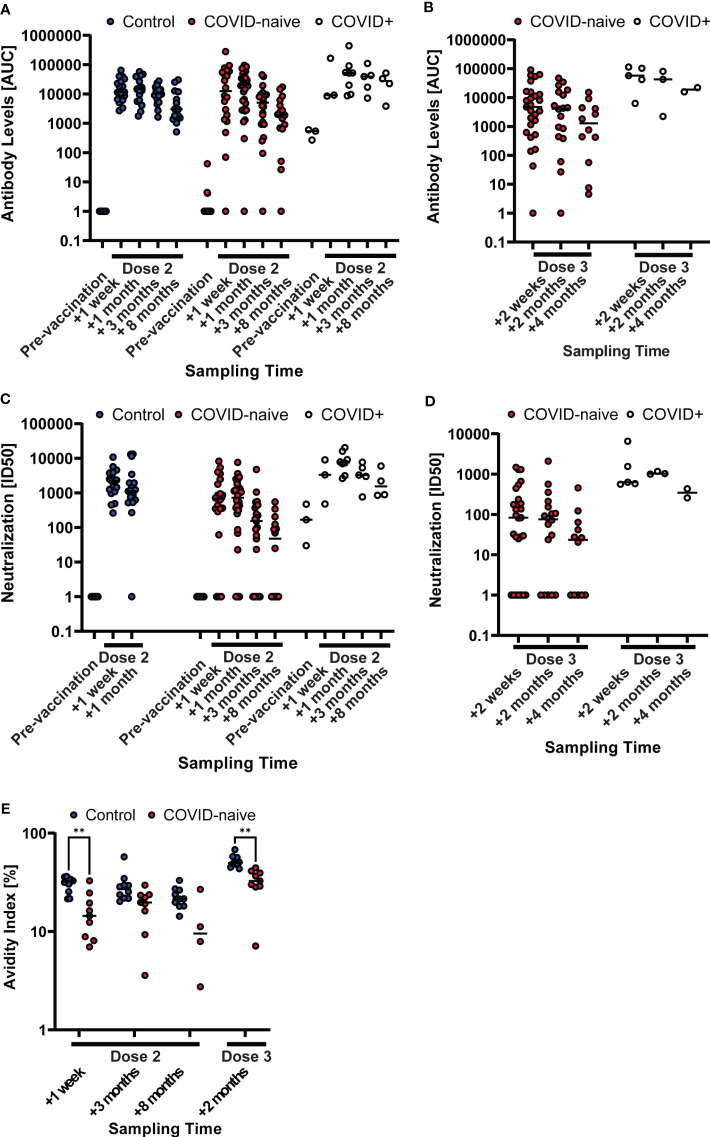
Generally impaired humoral immune response in COVID-naïve multiple sclerosis (MS) patients after vaccination. Longitudinal SARS-CoV-2 spike-specific antibody levels after two **(A)** and three **(B)** vaccine doses, and SARS-CoV-2-pseudotyped lentivirus neutralization capability after two **(C)** and three **(D)** vaccine doses of the non-MS control group and patient groups, the latter differentiated between patients that were infected with COVID-19 prior to vaccination (COVID+) and COVID-naïve patients. Antibody levels are described as the area under the curve (AUC) of a patient serum dilution series. Neutralization capability is described as the serum dilution required to reduce the viral infection by 50% (ID50). The horizontal, dashed line displays the reference value of a low-neutralizing control serum sample. Sample points, whose neutralizing capability or antibody levels were below detection have been set to 1 for illustrative purposes. **(E)** Longitudinal antibody avidity of a subset (n = 10) of the non-MS control and COVID-naïve patient groups. ** p<0.01.

We assessed circulating SARS-CoV-2 binding (**Figures 2A, B**) and neutralizing (**Figures 2C, D**) antibody levels to evaluate the response to the BNT162b2 vaccine. We could demonstrate that two doses of BNT162b2 induced similar levels of S-binding antibodies in SARS-CoV-2 naïve patients as in healthy individuals, and that this similarity remained up until eight months after the second dose (**Figure 2A**). Consistently, two doses of BNT162b2 during treatment interruption induced similar levels of neutralizing antibodies against the SARS-CoV-2 S-pseudotyped or authentic virus in patients and in matched control subjects (**Figure 2A**; **Supplementary Figure 2**). These data demonstrated that the time of treatment interruption had been sufficient to allow for efficient development of S-binding antibodies after a two-dose regimen of BNT162b2. In contrast, we found that the avidity of elicited S-binding IgG was lower in COVID-naïve patients than in healthy controls at one week (1.9-fold, p = 0.003), three months (1.6-fold), and eight months (1.8-fold) after two vaccine doses (**Figure 2E**).

Treatment with RTX was resumed for all patients four weeks after the second dose of BNT162b2. A third vaccine dose was given after an additional seven months. In contrast to the strong response in healthy individuals (17, 18), we found that a third dose in patients was inefficient to significantly boost antibody-mediated responses on a group level (**Figures 2B, D**). A third dose led to an increase in avidity of circulating S-specific IgG in both control and patient groups, but the patient’s IgG were of lower (1.6-fold, p = 0.001) avidity than those of the healthy controls (**Figure 2E**). Finally, patients who had been infected with SARS-CoV2 during RTX treatment interruption, but before vaccination, responded strongly to two doses of BNT162b2 (**Figures 2A, C**). Since we wanted to study the de-facto vaccine effect, COVID-experienced patients were excluded from further analysis.

### A low response to primary vaccination pre-disposes MS patients to low or absent response to a consecutive booster dose after continuation of RTX treatment

**Figure 3:**
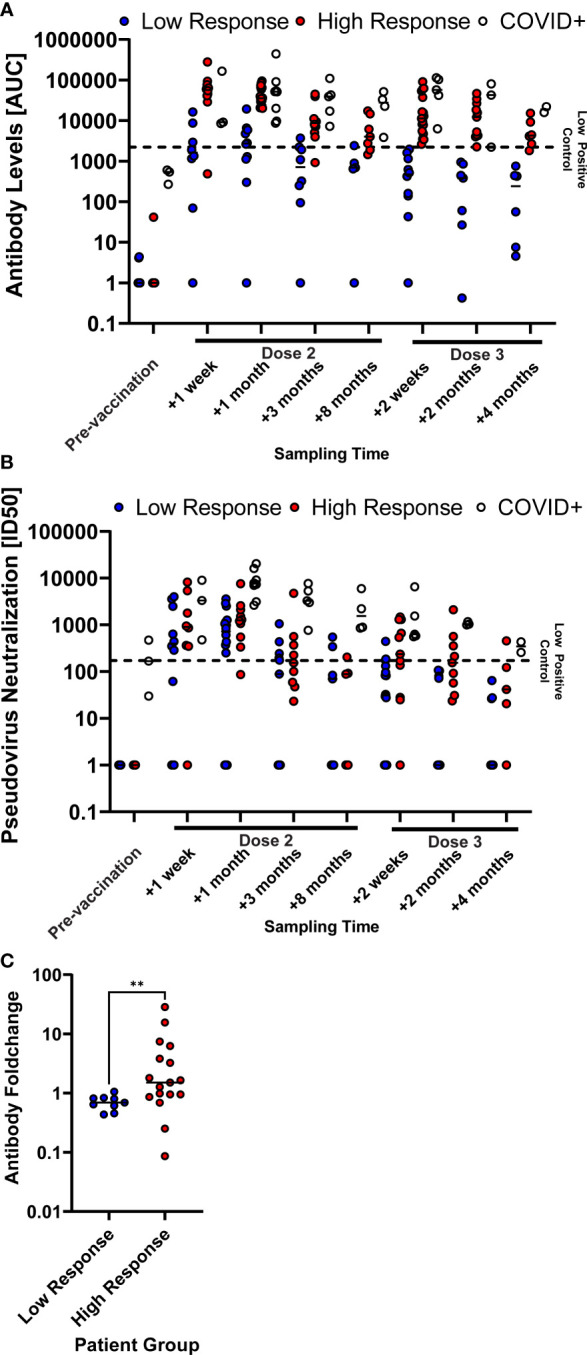
COVID-naïve multiple sclerosis (MS) patients with high SARS-CoV-2 antibody levels after the second vaccine dose showed a consistently high humoral immune response. SARS-CoV-2 spike-specific antibody levels **(A)** and SARS-CoV-2-pseudotyped lentivirus neutralization capability **(B)** of the patient groups, differentiating between patients that were infected with COVID-19 prior to vaccination (COVID+) and COVID naïve patients. COVID naïve patients were further grouped according to if their antibody levels were higher (High Response) or lower (Low Response) than the low-neutralizing control serum sample one week after the third vaccine dose. Antibody levels are described as the area under the curve (AUC) of a patient serum dilution series. Neutralization capability is described as the serum dilution required to reduce the viral infection by 50% (ID50). The horizontal, dashed line displays the reference value of a low-neutralizing control serum sample. Samples points, whose neutralizing capability or antibody levels were below detection have been set to 1 for illustrative purposes. **(C)** Anti-S IgG level foldchange between two weeks after and the last available time point (four to eight months) before the third vaccine dose of COVID-naïve MS patients. ** p < 0.01.

We noted that the variance of S-binding IgG was greater among patients compared to healthy controls. We therefore divided patients into two groups, low and high responders, based on the levels of circulating anti-S IgG after the third dose of BNT162b2. The median difference in levels of circulating anti-S IgG between the two subgroups was 36-fold (p = 0.007) at one week after the third dose. When analyzing these individuals retrospectively, we found that the low responder group had consistently lower levels of anti-S IgG in circulation after the second dose of BNT162b2 (p = 0.04 for one week after second dose, p = 0.03 for three months after second dose, **Figure 3A**). This was also reflected in lower levels of circulating SARS-CoV-2 neutralizing antibodies throughout all time points, albeit not significantly (**Figure 3B**). Importantly, we found that the boost effect of the third dose (fold-change) was significantly lower in the low responder group (p = 0.006, **Figure 3C**).

### Characterization of vaccine-induced B cells in MS patients after RTX treatment interruption

**Figure 4:**
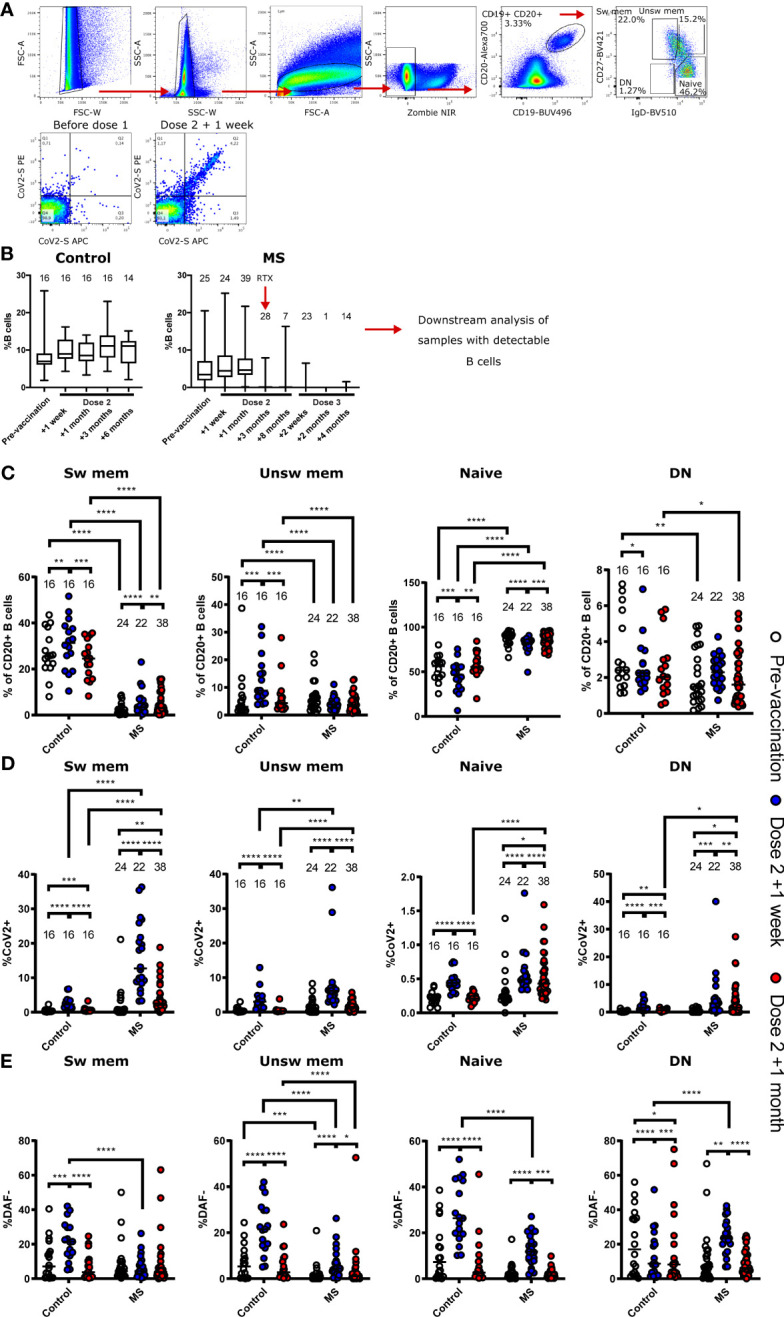
Flow cytometric characterisation of B cells from non- multiple sclerosis (MS) controls and MS patients. **(A)** Representative gating strategy for the final identification of switched memory (sw mem), unswitched memory (unsw mem), naïve, and double negative (DN) B cells. Definition of SARS-CoV2-specific B cells. **(B)** Longitudinal frequencies of B cells in non-MS controls and patients. Number of individuals are indicated above the respective box plots. Timepoint of re-initiated treatment with rituximab (RTX) is indicated in the patient group. **(C–E)** B cell frequencies in non-MS controls and patients before vaccination, one week and one month after the second vaccine dose. **(C)** Frequencies of sw mem, unsw mem, naïve, and DN B cells. **(D)** Frequencies of SARS-CoV2 specific B cells within the indicated subpopulations. **(E)** Frequencies of DAF- cells of each B cell subtype. * p<0.05; ** p<0.01; *** p<0.001; **** p<0.0001.

We performed flow cytometric analysis of the circulating B cell compartment of patients and healthy control subjects to investigate if the low response in certain individuals could be due to differential B cell activation after treatment interruption and two BNT162b2 doses. Consistent with the long interval of RTX treatment interruption, we found a similar frequency of circulating total B cells (CD19^+^) in patients as in healthy controls up until four weeks post two BNT162b2 doses (**Figures 4A, B**). Accordingly, RTX treatment re-initiation four weeks after the second BNT162b2 dose led to efficient depletion of the peripheral B cell compartment in patients (**Figure 4B**). We then assessed the distribution of switched memory B cells (CD19^+^CD20^+^CD27^+^IgD^-^), unswitched memory B cells (CD19^+^CD20^+^CD27^+^IgD^+^), naïve B cells (CD19^+^CD20^+^CD27^-^IgD^+^), and double negative (DN) B cells (CD19^+^CD20^+^CD27^-^IgD^-^). The frequencies of these populations in the control group were similar to previously reported findings (19), whereas the MS patients had reconstituted a predominantly naïve repertoire during RTX treatment interruption (**Figure 4C**).

We further dissected the peripheral B cell compartment based on B cell expression of an S-specific B cell receptor (BCR). This was done to determine if prior RTX treatment had an impact on vaccine-induced responses in patients. We found significantly elevated levels of S-specific switched memory B cells in patients one week and one month after the second BNT162b2 dose, as compared to healthy controls at the same timepoints (**Figure 4D**). A similar difference was noted for S-specific unswitched memory B cells and we could also demonstrate that patients had a higher frequency of antigen-specific DN B cells than healthy controls at four weeks post a second BNT162b2 dose (**Figure 4D**). We have previously shown that B cells downregulate the complement regulatory protein DAF upon BCR engagement, and that DN B cells in circulation may reflect ongoing B cell responses in lymphoid organs (19, 20). We found that patients had increased frequencies of DAF^-^ cells in all B cell populations except for switched memory B cells one week after the second dose (**Figure 4E**). In comparison, the control individuals demonstrated increased levels of DAF^-^ B cells in all populations except DN B cells.

### SARS-CoV-2 specific DN B cell levels one month after the second vaccine dose correlate with high antibody response and virus neutralization after the third dose

**Figure 5:**
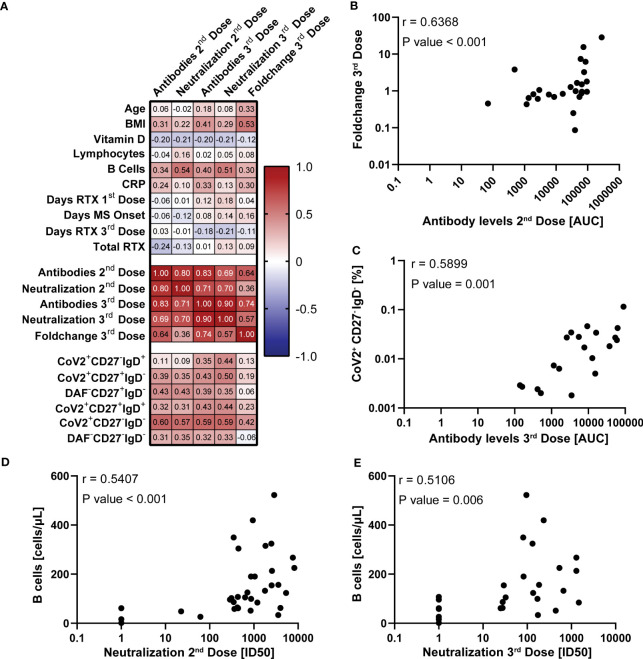
Correlation of vaccine response with demographic and clinical parameters in COVID-19 naïve multiple sclerosis (MS) patients. **(A)** Spearman correlation matrix between the SARS-CoV-2 spike-specific antibody levels together (AUC) with pseudotyped lentivirus neutralization capability (ID50) and multiple experimental and clinical parameters. Corresponding complete correlation matrix and P values are displayed in **Figures S3** and **S4**, respectively. Chosen parameters are listed in the Statistical Analysis subsection of the Methods part. **(B–E)** Spearman correlation of SARS-CoV-2 spike-specific antibody levels one week after the second vaccine dose and foldchange of SARS-CoV-2 spike-specific antibody levels after and before third vaccine dose **(B)**, SARS-CoV-2 spike-specific antibody levels one week after the third vaccine dose and SARS-CoV-2^+^CD27^-^IgD^-^ B cell levels **(C)**, total B cell levels before the first vaccine dose and SARS-CoV-2-pseudotyped lentivirus neutralization capability one week after the second **(D)** and third dose **(E)**. The analysis results are displayed in the upper left.

We next did a quantitative correlation analysis of the serological and clinical parameters, as well as frequencies of different B cell populations (**Figure 5**; **Supplementary Figure 3**). This was done to better understand if there were parameters that could predict the antibody response magnitude or quality after vaccination of COVID-naïve MS patients on RTX treatment interruption.

We found that BMI, but no other demographic factors, had a strong correlation with the fold-increase of S-specific IgG before and after a third vaccine dose (Foldchange 3^rd^ Dose) (**Figure 5A**). Additionally, we found that levels of circulating anti-S IgG one week after a second dose correlated with the fold-increase after a third vaccine dose after re-initiation of RTX treatment (**Figure 5B**). Of the clinical parameters, the total B cell count before the first vaccination dose correlated with the neutralization capability after the second and third vaccine doses (**Figures 5D, E**). Out of all B cell populations, the SARS-CoV-2 spike-protein specific DN B cell frequencies one month after the second vaccine dose displayed the highest correlation with the antibody levels and neutralization capability after the second and third vaccine doses (**Figures 5A, C**). In summary, these findings add to the observation that circulating anti-S IgG after a second dose of BNT162b2 has predictive value for the boost effect after RTX treatment re-initiation of MS patients.

## Discussion

In this study we investigated humoral immune responses to SARS-CoV-2 vaccination in RTX-treated MS patients during interruption and subsequent re-initiation of treatment. We observed that MS patients with previous COVID infection had a strong response to both second and third vaccine doses, comparable to the non-MS control group. These data demonstrate that SARS-CoV-2 infection of MS patients on RTX treatment interruption generates a strong immunological memory that can be re-stimulated by vaccination, similarly to healthy individuals (21). However, COVID-naïve MS patients displayed high variability in antibody levels after both second and third vaccine doses, showing similar or lower levels as COVID-experienced MS patients and non-MS controls. Due to our study participant number being limited to 42 eligible individuals, we could not show any differences in vaccine response due to sex, age, comorbidities, or additional medication. Moreover, experiments examining the plasma blast repertoire could complement the S-specific antibody binding and neutralization results obtained by this study. Nevertheless, our results indicate highly variable vaccine responses within the overall COVID-naïve MS patient group and offer first laboratory correlates describing this variance.

Through B cell phenotype characterization, we could show that MS patients exhibited a significantly higher naïve B cell population and consequently lower memory (CD27^+^) subsets than the control group before and after SARS-CoV-2 vaccination. These findings coincide with previous reports of B cell reconstitution after RTX treatment, where the B cell populations were limited to immature, transitional, and mature naïve B cells, with low levels of memory subsets (22, 23). We additionally show that both MS patients and controls developed a robust SARS-CoV-2 S-specific memory B cell population upon vaccination. Although naïve B cell population frequencies were reported to be a general predictor for SARS-CoV-2 vaccine response in immunocompromised individuals (24), this parameter alone could not explain the highly variable vaccine response of our COVID-naïve MS patients. Strikingly, SARS-CoV-2^+^ DN B cell frequencies were consistently higher in MS patients than in controls after vaccination. We show that this B cell subset correlates significantly with the antibody and neutralization levels after the second and third vaccine dose. To our knowledge, there has been no report investigating the function of DN B cells in RTX-treated MS patients. This B cell subset is inflated in patients with different chronic inflammatory conditions and after vaccination against influenza and tick-borne encephalitis virus (25, 26). In addition, DN B cells are shown to transcriptionally cluster with naïve, memory B cells, and plasmablasts, suggesting an extra-follicular maturation pathway (26).

Germinal centre (GC) B cells express low levels of DAF, a phenomenon that we also observed in circulating B cells during acute Hantavirus infection (20). We suppose that the increase of DAF^-^ B cells might reflect a robust GC response, since GC B cells downregulate their DAF expression. Indeed, the complement regulation in GCs has a crucial role in positive B cell selection and therefore antigen-induced antibody production (27). Another factor that may be indicative of the GC activity is the avidity of elicited antibodies. Here, we found that MS patients had an overall lower avidity compared to the control group. The lower antibody avidity in MS patients in combination with the decreased expansion of DAF^-^ sw mem B cells might suggest that the B cell affinity maturation in the GC could be impaired as compared to the control group. Previous reports show that GC activity after RTX is negatively affected (28, 29). To date, however, there are no long-term effect studies on the impact of RTX on GC formation in MS individuals after treatment interruption. Our T cell analysis (**Supplementary Figure 4**) showed a comparable trend in T cell populations between the control and patient groups after vaccination. Consistently, previous vaccination studies indicate the preservation of T cell levels and responses in anti-CD20 treated MS individuals (30).

In summary, our findings highlight the heterogeneity of humoral immune responses to SARS-CoV-2 mRNA vaccination in RTX-treated MS patients and provide factors predictive of the response. The high vaccine response in a fraction of COVID-naïve MS patients hints towards the survival of SARS-CoV-2-specific memory B cells after re-initiation of RTX treatment. This may be due to differential depletion of different B cell populations within secondary lymphoid organs by RTX (29). In addition, we show that SARS-CoV-2^+^ DN B cell frequencies one month after the second vaccine dose significantly correlate with a higher antibody response to the second and third vaccine doses. Importantly, S-binding antibody levels after the second vaccine dose could predict efficient immune responses to subsequent vaccine doses during ongoing RTX treatment. Taken together, this could aid the decision to antedate the third vaccine dose by several months while postponing re-initiation of RTX for individuals with poor immune responses to the first two vaccine doses. Lastly, our findings provide possible leads to research crucial aspects of vaccine responses in RTX-treated MS patients. The investigation of DN B cells and their potential extra-follicular maturation could provide deeper insight into alternative antigenic stimulation of B cells in these patients. Furthermore, the possible long-term impairment of GC activity in B-cell depleted MS patients needs further examination to understand and counteract their attenuated vaccine response.

## Data availability statement

The datasets for this article are not publicly available due to concerns regarding participant/patient anonymity. Requests to access the datasets should be directed to the corresponding author.

## Ethics statement

The studies involving human participants were reviewed and approved by Swedish Ethical Review Authority. The patients/participants provided their written informed consent to participate in this study.

## Author contributions

RG: Designed and performed experiments, conducted analyses, and wrote the manuscript. AD: Designed, performed, and analysed flow cytometric experiments, critically read and wrote the manuscript. PS, MF, CA and JN: Planned/conceived and designed the study, reviewed and edited the manuscript. PS: Responsible for recruitment of MS patients. CA and JN are principal investigators for the clinical trial and supervised sample and data collection. All authors contributed to the article and approved the submitted version.

## References

[1] Dobson R Giovannoni G. Multiple sclerosis – a review. Eur J Neurol (2019) 26(1):27–40. doi: 10.1111/ene.13819 30300457

[2] Ascherio A. Environmental factors in multiple sclerosis. Expert Rev Neurother (2013) 13(12 SUPPL.):3–9. doi: 10.1586/14737175.2013.865866 24289836

[3] Wolinsky JS Narayana PA O’Connor P Coyle PK Ford C Johnson K. Glatiramer acetate in primary progressive multiple sclerosis: results of a multinational, multicenter, double-blind, placebo-controlled trial. Ann Neurol (2007) 61(1):14–24. doi: 10.1002/ana.21079 17262850

[4] Hauser SL Waubant E Arnold DL Vollmer T Antel J Fox RJ. B-cell depletion with rituximab in relapsing–remitting multiple sclerosis. N Engl J Med (2008) 358(7):676–88. doi: 10.1056/NEJMoa0706383 18272891

[5] Svenningsson A Frisell T Burman J Salzer J Fink K Hallberg S. Safety and efficacy of rituximab versus dimethyl fumarate in patients with relapsing-remitting multiple sclerosis or clinically isolated syndrome in Sweden: a rater-blinded, phase 3, randomised controlled trial. Lancet Neurol (2022) 21(8):693–703. doi: 10.1016/S1474-4422(22)00209-5 35841908

[6] Sellebjerg F Blinkenberg M Sorensen PS. Anti-CD20 monoclonal antibodies for relapsing and progressive multiple sclerosis. CNS Drugs (2020) 34(3):269–80. doi: 10.1007/s40263-020-00704-w 31994023

[7] Smith TE Madhavan M Gratch D Patel A Saha V Sammarco C. Risk of COVID-19 infection and severe disease in MS patients on different disease-modifying therapies. Mult Scler Relat Disord (2022) 60. doi: 10.1016/j.msard.2022.103735 PMC8915504 35398713

[8] Sormani MP De Rossi N Schiavetti I Carmisciano K Cordioli C Moiola L. Disease-modifying therapies and coronavirus disease 2019 severity in multiple sclerosis. Ann Neurol (2021) 89(4):780–9. doi: 10.1002/ana.26028 PMC8013440 33480077

[9] Luna G Alping P Burman J Fink K Fogdell-Hahn A Gunnarsson M. Infection risks among patients with multiple sclerosis treated with fingolimod, natalizumab, rituximab, and injectable therapies. JAMA Neurol (2020) 77(2):184–91. doi: 10.1001/jamaneurol.2019.3365 PMC6784753 31589278

[10] Spelman T Forsberg L McKay K Glaser A Hillert J. Increased rate of hospitalisation for COVID-19 among rituximab-treated multiple sclerosis patients: a study of the Swedish multiple sclerosis registry. Mult Scler J (2022) 28(7):1051–9. doi: 10.1177/13524585211026272 34212816

[11] Achiron A Mandel M Dreyer-Alster S Harari G Dolev M Menascu S. Humoral immune response in multiple sclerosis patients following PfizerBNT162b2 COVID19 vaccination: up to 6 months cross-sectional study. J Neuroimmunol (2021) 361. doi: 10.1016/j.jneuroim.2021.577746 PMC8500842 34655991

[12] Tolf A Wiberg A Müller M Nazir FH Pavlovic I Laurén I. Factors associated with serological response to SARS-CoV-2 vaccination in patients with multiple sclerosis treated with rituximab. JAMA Netw Open (2022) 5(5):e2211497. doi: 10.1001/jamanetworkopen.2022.11497 35544139 PMC9096596

[13] Kelly H Sokola B Abboud H. Safety and efficacy of COVID-19 vaccines in multiple sclerosis patients. J Neuroimmunol (2021) 356. doi: 10.1016/j.jneuroim.2021.577599 PMC8095041 34000472

[14] MS-Sällskapet S. Rekommendationer gällande vaccination vid MS. Available at: https://www.mssallskapet.se/wp-content/uploads/2020/02/Rekommendationer-g%C3%A4llande-vaccination-vid-MS-.pdf.

[15] Kurtzke JF. Rating neurologic impairment in multiple sclerosis: an expanded disability status scale (EDSS). Neurology (1983) 33(11):1444–4. doi: 10.1212/WNL.33.11.1444 6685237

[16] Byström JW Vikström L Rosendal E Gröning R Gwon YD Nilsson E. Serological assessment of SARS-CoV-2 exposure in northern Sweden by the use of at-home sampling to meet geographical challenges in rural regions. medRxiv (2022) 28. doi: 10.2807/1560-7917.ES.2023.28.13.2200432 PMC10064644 36995373

[17] Barda N Dagan N Cohen C Hernán M Lipsitch M Kohane IS. Effectiveness of a third dose of the BNT162b2 mRNA COVID-19 vaccine for preventing severe outcomes in Israel: an observational study. Lancet (2021) 398(10316):2093–100. doi: 10.1016/S0140-6736(21)02249-2 PMC8555967 34756184

[18] Munro APS Janani L Cornelius V Aley PK Babbage G Baxter D. Safety and immunogenicity of seven COVID-19 vaccines as a third dose (booster) following two doses of ChAdOx1 nCov-19 or BNT162b2 in the UK (COV-BOOST): a blinded, multicentre, randomised, controlled, phase 2 trial. Lancet (2021) 398(10318):2258–76. doi: 10.1016/S0140-6736(21)02717-3 PMC8639161 34863358

[19] Kerkman PF Dernstedt A Tadala L Mittler E Dannborg M Sundling C. Generation of plasma cells and CD27–IgD– B cells during hantavirus infection is associated with distinct pathological findings. Clin Transl Immunol (2021) 10(7). doi: 10.1002/cti2.1313 PMC8275445 34277007

[20] Dernstedt A Leidig J Holm A Kerkman PF Mjösberg J Ahlm C. Regulation of decay accelerating factor primes human germinal center B cells for phagocytosis. Front Immunol (2021) 11. doi: 10.3389/fimmu.2020.599647 PMC7813799 33469456

[21] Ali H Alahmad B Al-Shammari AA Alterki A Hammad M Cherian P. Previous COVID-19 infection and antibody levels after vaccination. Front Public Heal (2021) 9. doi: 10.3389/fpubh.2021.778243 PMC8671167 34926392

[22] Roll P Palanichamy A Kneitz C Dorner T Tony HP. Regeneration of B cell subsets after transient B cell depletion using anti-CD20 antibodies in rheumatoid arthritis. Arthritis Rheum (2006) 54(8):2377–86. doi: 10.1002/art.22019 16869000

[23] Colucci M Carsetti R Cascioli S Casiraghi F Perna A Ravà L. B cell reconstitution after rituximab treatment in idiopathic nephrotic syndrome. J Am Soc Nephrol (2016) 27(6):1811–22. doi: 10.1681/ASN.2015050523 PMC4884116 26567244

[24] Schulz E Hodl I Forstner P Hatzl S Sareban N Moritz M. CD19+IgD+CD27- naïve B cells as predictors of humoral response to COVID 19 mRNA vaccination in immunocompromised patients. Front Immunol (2021) 12:803742. doi: 10.3389/fimmu.2021.803742 34950155 PMC8688243

[25] Jenks SA Cashman KS Zumaquero E Marigorta UM Pater AV Wang X. Distinct effector B cells induced by unregulated toll-like receptor 7 contribute to pathogenic responses in systemic lupus erythematosus. Immunity (2018) 49(4):725–739.e6. doi: 10.1016/j.immuni.2018.08.015 30314758 PMC6217820

[26] Ruschil C Gabernet G Lepennetier G Heumos S Kaminski M Hracsko Z. Specific induction of double negative B cells during protective and pathogenic immune responses. Front Immunol (2020) 11:1–15. doi: 10.3389/fimmu.2020.606338 PMC7775384 33391273

[27] Cumpelik A Heja D Hu Y Varano G Ordikhani F Roberto MP. Dynamic regulation of B cell complement signaling is integral to germinal center responses. Nat Immunol (2021) 22(6):757–68. doi: 10.1038/s41590-021-00926-0 PMC8297556 34031614

[28] Carubbi F Cipriani P Marrelli A Di Benedetto P Ruscitti P Berardicurti O. Efficacy and safety of rituximab treatment in early primary Sjögren’s syndrome: a prospective, multi-center, follow-up study. Arthritis Res Ther (2013) 15(5). doi: 10.1186/ar4359 PMC3979092 24286296

[29] Damato V Theorell J Al-Diwani A Kienzler AK Makuch M Sun B. Rituximab abrogates aquaporin-4-specific germinal center activity in patients with neuromyelitis optica spectrum disorders. Proc Natl Acad Sci USA (2022) 119(24):e2121804119. doi: 10.1073/pnas.2121804119 35666871 PMC9214492

[30] Schwarz T Otto C Jones TC Pasche F Schindler P Niederschweiberer M. Preserved T cell responses to SARS-CoV-2 in anti-CD20 treated multiple sclerosis. Mult Scler J (2022) 28(7):1041–50. doi: 10.1177/13524585221094478 PMC9131414 35575234

