# Supplementary material for: Immune response to SARS-CoV-2 mRNA vaccination in multiple sclerosis patients after rituximab treatment interruption
Source: Front Immunol. 2023 Jul 27;14:1219560. doi: 10.3389/fimmu.2023.1219560 (PMC10413123; doi:10.3389/fimmu.2023.1219560)
Supplement: Supplementary file 1 [file DataSheet_1.docx]

***Supplementary Material***

**Immune response to SARS-CoV-2 mRNA vaccination in multiple sclerosis patients after rituximab treatment interruption**

**Remigius Gröning^1^, Andy Dernstedt^1^, Clas Ahlm^1^, Johan Normark^1-3^, Peter Sundström^1,4^, and Mattias NE Forsell^1*^**

^1^Department of Clinical Microbiology, Umeå University, Umeå, Sweden
^2^Molecular Infection Medicine Sweden (MIMS), Umeå University, Umeå, Sweden
^3^Wallenberg Centre for Molecular Medicine (WCMM), Umeå University, Umeå, Sweden
^4^Department of Clinical Sciences, Neurosciences, Umeå University, Umeå, Sweden

***Correspondence:**Mattias NE Forsell
Department Clinical Microbiology, Umeå University
Målpunkt R NUS 90187 Umeå
[mattias.forsell@umu.se](mailto:mattias.forsell@umu.se)
+46 90 785 32 08

**
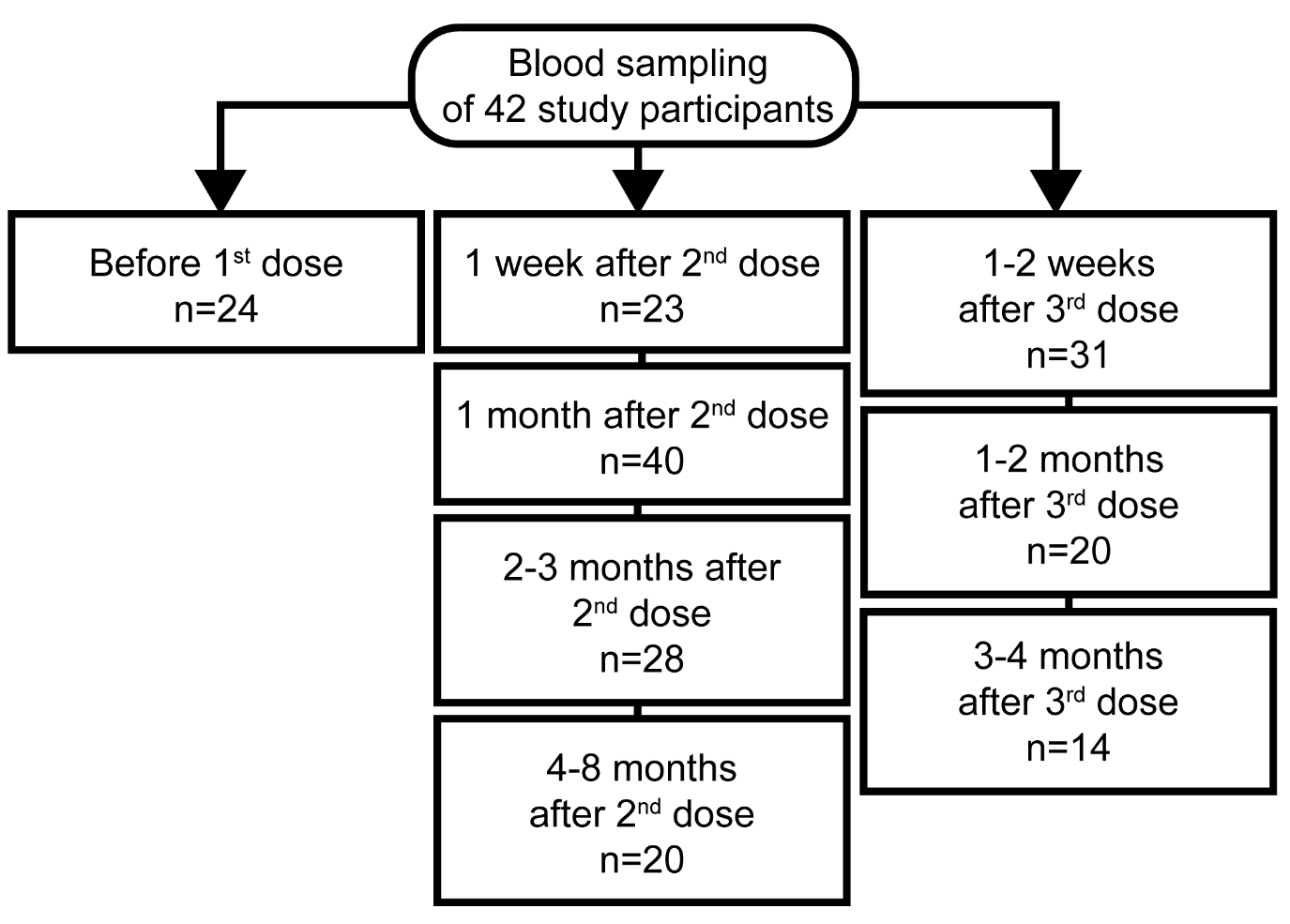
**

**Supplementary Figure 1: Blood sample time points and sample sizes of rituximab (RTX)-treated multiple sclerosis (MS) patients during SARS-CoV-2 vaccination.**

**
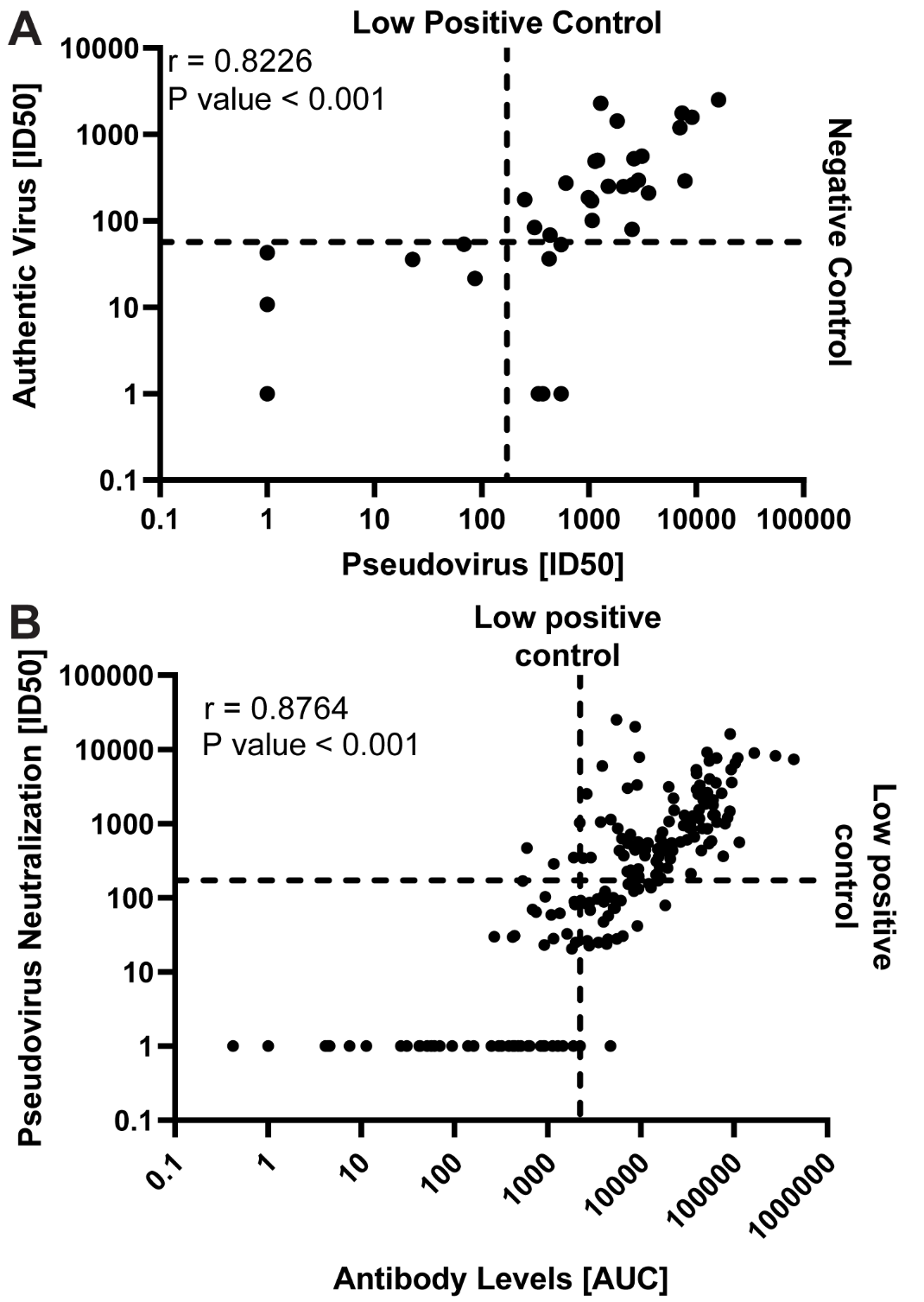
**

**Supplementary Figure 2: Correlation of authentic and pseudotyped SARS-CoV-2 virus neutralization.** Comparison of authentic and pseudotyped SARS-CoV-2 virus neutralization assay (A), as well as SARS-CoV-2 spike-specific antibody levels and pseudotyped virus neutralization capability (B) of MS patient samples. Sample points, whose neutralizing capability or antibody levels were below detection have been set to 1 for illustrative purposes. Statistical parameters in the upper left describe the results of a Spearman correlation analysis. (A) Plot of authentic SARS-CoV-2 virus against SARS-CoV-2-pseudotyped lentivirus neutralization capability, described as the serum dilution required to reduce the pseudovirus infection by 50% (ID50), for MS patient serum samples one month after the second vaccine dose. The vertical and horizontal lines display the reference values for a low neutralizing and negative control serum sample, respectively. (B) Plot of SARS-CoV-2 spike-specific antibody levels (AUC) against SARS-CoV-2-pseudotyped lentivirus neutralization capability (ID50) for MS patient samples. The vertical and horizontal lines display the reference value for a low neutralizing control serum sample.

**
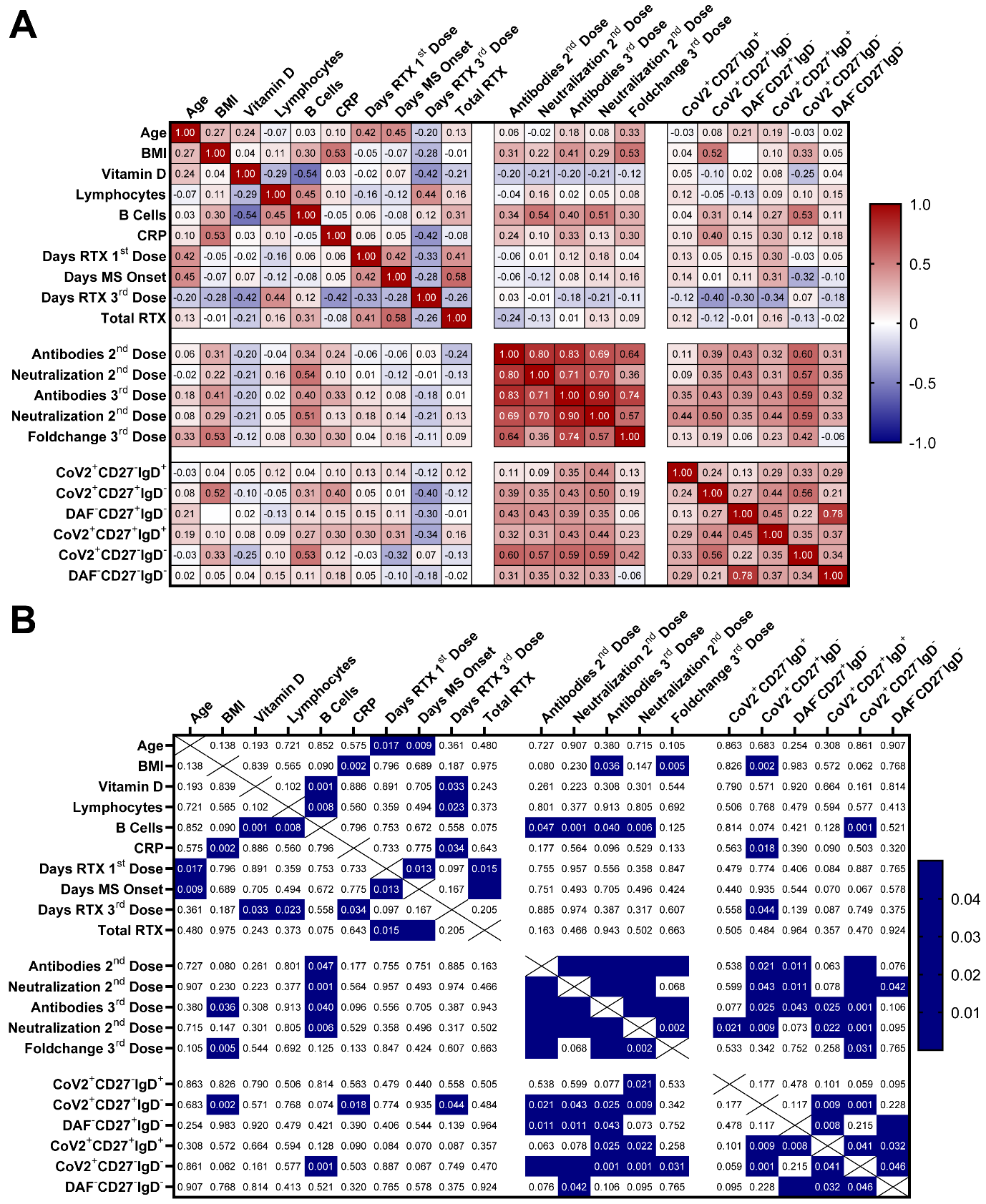
**

**Supplementary Figure 3: Complete correlation of multiple experimental and clinical parameters in COVID-19 naïve multiple sclerosis (MS) patients.** (A) Spearman correlation matrix between multiple experimental and clinical parameters of COVID-19 naïve MS patients. (B) P values of the corresponding spearman correlation matrix. Matrix cells with a P value below 0.05 were depicted in blue. Chosen parameters are listed in the data analysis part of the materials and methods section.

**
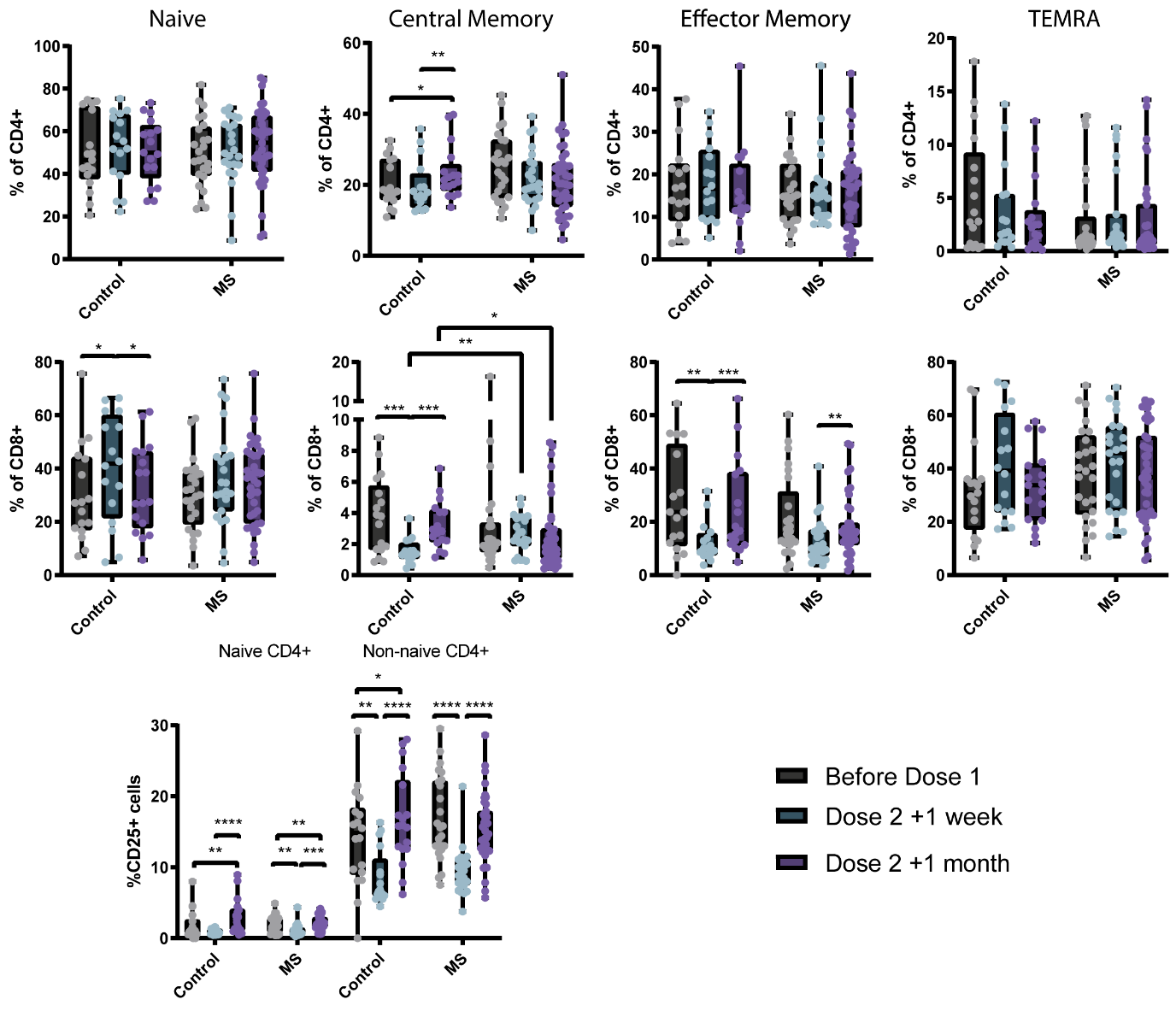
**

**Supplementary Figure 4:** **Frequencies of T cell populations based on their expression of CCR7 and CD45RA in non-multiple sclerosis (MS) controls and MS patients.** Displayed are naïve (CCR7^+^CD45RA^+^), central memory (CCR7^+^ CD45RA^-^), effector memory (CCR7^-^ CD45RA^-^), and terminally differentiated effectors (CCR7^-^ CD45RA^+^). Upper row: CD4^+^ T cells. Middle row: CD8^+^ T cells. Bottom row: %CD25^+^ naïve and non-naïve CD4^+^ T cells.
